# Findings from a Statewide Teleretina Diabetic Retinopathy Screening Program in Arkansas

**DOI:** 10.1155/2023/3233803

**Published:** 2023-03-24

**Authors:** Megan Shirey, Alexander Kwok, Holly Jenkins, Sami Uwaydat

**Affiliations:** ^1^University of Arkansas for Medical Sciences: Harvey and Bernice Jones Eye Institute, Little Rock, AR 72205, USA; ^2^University of Arkansas for Medical Sciences, Little Rock, AR 72205, USA

## Abstract

**Introduction:**

A significant proportion of diabetic patients in the United States do not present for annual dilated eye exams to monitor for signs of diabetic retinopathy (DR). The purpose of this study was to analyze the results of a statewide, multiclinic teleretina program designed to screen rural Arkansans for this sight-debilitating disease.

**Methods:**

Patients with diabetes seen at 10 primary care clinics across Arkansas were offered teleretinal-imaging services. Images were transmitted to the University of Arkansas for Medical Sciences' (UAMS) Harvey and Bernice Jones Eye Institute (JEI) for grading and recommendations for further treatment.

**Results:**

From February 2019 to May 2022, 668 patients underwent imaging; 645 images were deemed of sufficient quality to generate an interpretation. 541 patients had no evidence of DR, while 104 patients had some evidence of DR. 587 patients had no evidence of maculopathy, while 58 patients had some evidence of maculopathy on imaging. 246 patients had other pathology evident on imaging, with the most common being hypertensive retinopathy, glaucoma suspects, and cataracts. *Discussion*. In a rural, primary care setting, the JEI teleretina program identifies DR and other nondiabetic ocular pathologies, allowing for an appropriate triage for eye care for patients in a predominantly rural state.

## 1. Introduction

Per the Center for Disease Control (CDC) 2020 diabetes statistics report, 34.2 million people, or 10.5% of the United States population, have diabetes. However, an estimated 7.3 million of this group are currently undiagnosed with the disease [1]. A significant proportion of patients in the United States of America (USA) with diabetes do not present for annual dilated eye exams to monitor for early retinal disease. According to the most recent estimates from the CDC, only 62.3% of patients over the age of 18 with diabetes received an eye exam within the last year [2]. Failure to detect early changes in diabetic retinopathy (DR) ultimately increases the proportion of patients with significant vision loss secondary to advanced DR.

Diabetic retinopathy is classified in stages based on the severity of retinal hemorrhages, macular edema, and/or the absence or presence of neovascularization. Nonproliferative (no neovascularization) disease can present as mild, moderate, or severe. Proliferative (presence of neovascularization) disease can present with vision loss from retinal detachments or vitreous hemorrhage. Any stage of DR has the potential to present with diabetic macular edema (DME), and this can significantly impact a patient's visual acuity. Treatment for DME and proliferative retinopathy consists of intravitreal antivascular endothelial growth factor (VEGF) injections, panretinal photocoagulation, and, in advanced cases, retinal surgery to treat associated vitreous hemorrhage and retinal detachment. The potential to preserve useful vision is highest when the treatment is initiated prior to the onset of irreversible retinal damage, hence the incentive for screening diabetic patients to detect the earlier stages of DR.

In the USA, diabetes is currently the leading cause of new cases of blindness among 18-64 year-olds [1]. It is also predicted that by 2050, the incidence of DR will increase by 72% and vision impairment and blindness by 150% without effective interventions [3, 4]. The US Department of Health and Human Services reports that only 62.3% of adults diagnosed with diabetes had an eye exam within the past year [2, 5]. Numerous contributing factors to this high noncompliance rate have been reported in the literature [3]. Many of these challenges are faced by those located in rural communities. Arkansas is home to a large rural population. According to 2019 population estimates, 41% of the state's population lives in rural countries. This contrasts with the 14% of the overall United States population who live in rural counties [6].

Teleretinal imaging can help fill the large gap of diabetics missing annual eye exams. The teleretina program at the Harvey and Bernice Jones Eye Institute (JEI) at the University of Arkansas for Medical Sciences (UAMS) aims to decrease the percentage of Arkansans with permanent vision loss due to diabetes by significantly increasing the number of diabetic patients who receive a retinal evaluation. Additionally, these retinal images can also detect other eye diseases such as glaucoma and age-related macular degeneration. Our goal was to evaluate the DR teleretina screening program in patients who live in rural and/or underserved portions of Arkansas.

## 2. Methods

UAMS oversees primary care clinics that serve the predominantly rural population across the state of Arkansas. Ten of these clinics, located in the East, Northwest, North Central, Northeast, West, South Central, and Southwest regions as well as three clinic areas within the Central Arkansas region were each provided with a nonmydriatic camera, either a newer TRC-NW400 (Topcon Healthcare, Oakland, New Jersey) model or an older TRC-NW300 (Topcon Healthcare, Oakland, New Jersey) model. The clinic staff was trained in camera operation. Patients seen in these clinics who identified themselves as diabetic or were diagnosed with diabetes had a fundus picture taken as part of their work up. The pictures were uploaded to ZEISS FORUMÒ, an eyecare data management system that stores images and provides clinical tools for the assessment of retinal and other ocular diseases, and patient information was collected on a Microsoft Excel spreadsheet. Two eye care professionals (one ophthalmologist and one optometrist) located at UAMS' main campus in Little Rock, Arkansas, interpreted the fundus photographs using a standard interpretation report for each patient. It included the quality of the fundus photos (adequate, blurry, missing field), the retinopathy and maculopathy stage for each eye, and a separate section for any additional ocular pathologies noted. Finally, a recommendation was listed to either repeat imaging in one year or to refer to an eye care specialist for evaluation.

This study was a retrospective chart review of adult patients (≥18 years) evaluated at any one of the clinics between February 2019 and May 2022 who had a fundus picture taken during their visit. The study was approved by the UAMS Institutional Review Board; as routine fundus examination is an established component of screening for DR in patients with diabetes and the study involved only a retrospective review using existing data, a waiver for the informed consent process was granted. Additionally, the study was conducted in accordance with the tenets of the Declaration of Helsinki.

## 3. Results

### 3.1. Population Data

**Figure 1:**
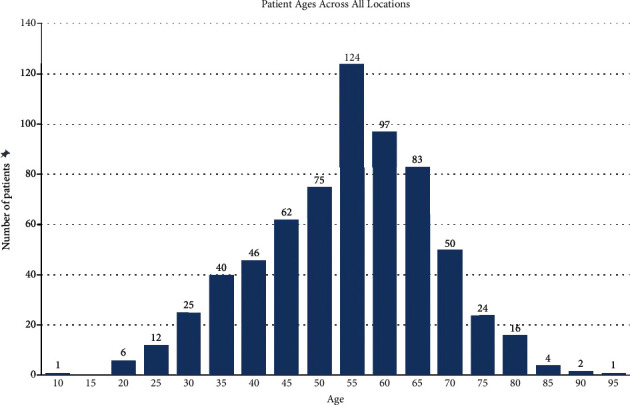
Age distribution of patients screened.

A table of descriptive statistics is provided in Table 1. Six hundred sixty-eight total patients were screened. Of these, 23 (3.4%) had poor images that could not be interpreted, leaving a total of 645 with complete reports. Of the 668 teleretina patients, 356 (53%) were female and 312 (47%) were male. The average age was 56.15 years, with a standard deviation of 13.29 years. Stratified by 5-year age groups, those between the ages of 55 and 60 were the most prevalent, making up 18.56% of the total patients screened (Figure 1).

**Figure 2:**
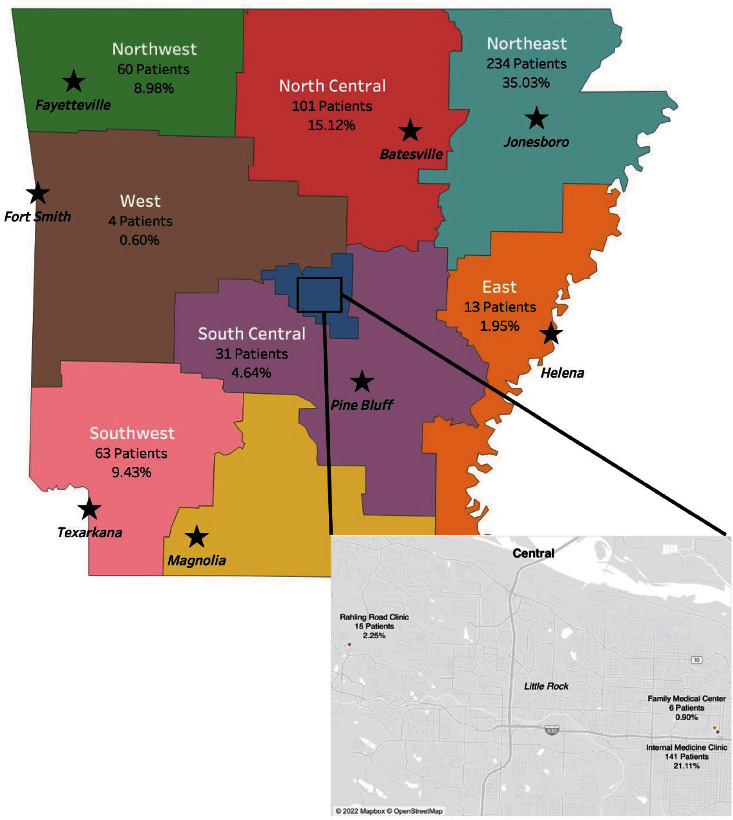
Arkansas state map depicting UAMS primary care clinics across the state with the number of patients screened for diabetic retinopathy at each location. The inset map depicts the three clinic sites located in the Central Arkansas region.

Broken down by clinical site, the number of patients screened was as follows: 60 patients (8.98%) from the Northwest region, 101 patients (15.12%) from the North Central region, 234 patients (35.03%) from the Northeast region, 4 patients (0.60%) from the West region, 13 patients (1.95%) from the East region, 63 patients (9.43%) from the Southwest region, 31 patients (4.64%) from the South Central region, 15 patients (2.25%) from the Rahling Clinic, 6 patients (0.90%) from the Family Medical Center, and 141 patients (21.11%) from the Internal Medicine Clinic (Figure 2).

### 3.2. Race

**Figure 3:**
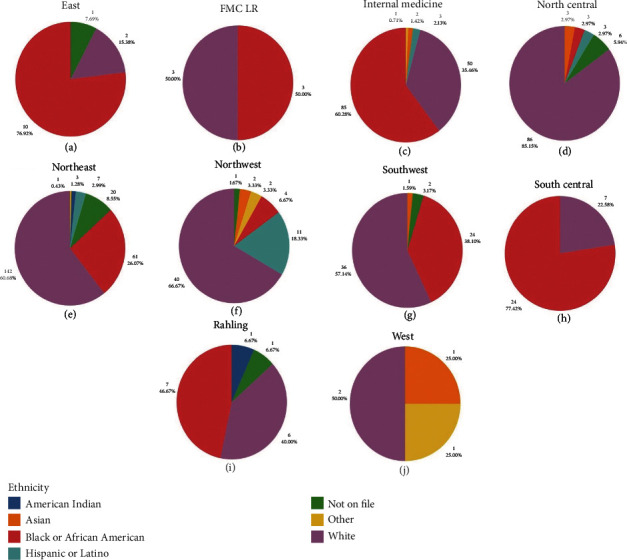
Graphs showing the distribution of race at each of the regional programs participating in the teleretina screening program: (a) East, (b) FMC LR, (c) Internal medicine, (d) North Central, (e) Northeast, (f) Northwest, (g) Southwest, (h) South Central, (i)Rahling, and (j) West. FMC LR: Family Medical Center Little Rock.

Of the 668 teleretina patients screened, 374 (56%) were White, 221 (33.1%) were Black or African American, 24 (3.6%) were Hispanic or Latino, 9 (1.3%) were Asian, 4 (0.6%) were Native American, 5 (0.7%) had their race listed as “other,” and 31 (4.6%) had a race/ethnicity that was indeterminable from their electronic medical record.  Figure 3 shows the racial breakdown of patients seen at each clinical site.

### 3.3. Diabetes Type

Of the 668 total patients screened, 647 (97%) had a diagnosis of type 2 diabetes, while the remaining 21 patients (3%) had a diagnosis of type 1 diabetes. Two hundred forty-four patients (37%) in the sample were insulin-dependent (Table 1).

### 3.4. Image Quality

Of the 668 total image sessions reviewed, 645 (96.6%) were of sufficient quality to allow for the interpretation. Of these, 467 (69.9%) were considered to be of adequate quality, while 178 (26.6%) sessions were either blurry, had a field missing, or both. These images were still given a diagnosis based on the views available. The remaining 23 image sessions were considered uninterpretable. The occurrence of each designation by the clinical site is detailed in Table 2.

### 3.5. Last Eye Exam

**Figure 4:**
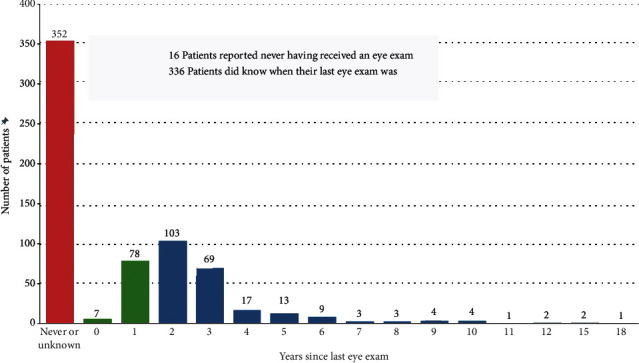
Bar graph depicting the number of patients by year since their last reported eye exam.

Of the 668 total patients screened, 352 patients (52.6%) reported not knowing when their last eye exam was or never having had an eye exam. Only 85 patients (12.7%) reported having an eye exam within a year or less. The remaining time frames are displayed in Figure 4.

### 3.6. Diabetic Retinopathy

Of the 668 teleretina reports collected, 645 were of sufficient quality to allow for interpretation. Within the subset of those with interpretable photographs, the majority of patients had type 2 diabetes (625 patients, 96.9%), while the remainder had type 1 diabetes (20 patients, 3.1%). Out of the interpretable images, 103 (16%) had evidence of DR. Of the type 1 patients, 8 (40%) had evidence of DR and were advised to schedule a comprehensive eye exam. Of those with type 2 diabetes, 95 (15%) had evidence of DR with the recommendation of scheduling a comprehensive eye exam (Table 3).

Retinopathy was categorized into five groups: no DR (R0), microaneurysm and/or hemorrhage (R1), venous beading and/or intraretinal microvascular abnormalities (IRMA) (R2), neovascularization of the optic disc (NVD), neovascularization elsewhere (NVE), and/or laser scars (R3), and retinal detachment (R4).

Of those with interpretable images, 541 patients (84.2%) were categorized as R0, while 104 patients (15.8%) had some degree of DR. Of these, 92 (14.3%) were categorized as R1, 4 (0.6%) were categorized as R2, and 6 (0.9%) were categorized as R3. None were categorized as R4.

Prevalence rates of DR by ethnicity for the sample population were as follows: 40% in other, 25% in American Indian patients, 19.2% in Black or African American patients, 16.7% in patients who did not have an ethnicity on file, 14.7% in White patients, 8.7% in Hispanic or Latino patients, and 0% in Asian patients (Table 4).

### 3.7. Maculopathy

Maculopathy was categorized into three groups: no diabetic maculopathy (M0), microaneurysm hemorrhage or exudate greater than one disc diameter from the center of the fovea (M1), and microaneurysm hemorrhage or exudate less than or equal to one disc diameter from the center of the fovea (M2).

Of the 645 interpretable photographs, 587 (91%) were classified as M0, 23 (3.6%) were classified as M1, and 35 (5.7%) were classified as M2.

### 3.8. Other Ocular Pathologies

Of the 645 total patients in the sample, 246 patients (38.1%) had other ocular pathologies detectable on fundus photography (Table 5). Forty-one patients presented with more than one other pathology. The most commonly detected condition was hypertensive retinopathy (71 patients), followed by glaucoma suspects (70 patients) and cataracts (67 patients). Other more common conditions identified included age-related macular degeneration, both nonexudative and exudative (17 patients), optic nerve edema, both unilateral or bilateral (17 patients), and choroidal nevi (14 patients). Additional conditions detected included macular scars, chorioretinal scars, Hollenhorst plaques, retinitis pigmentosa, optic nerve pallor, retinal detachment, venous occlusion/insufficiency, optic nerve hemorrhage, a macular granular deposits, macular hole, presumed ocular histoplasmosis syndrome, and pigment epithelial detachment.

Of those who presented with additional pathology, more than 50% were of an ethnicity other than White (Table 6).

## 4. Discussion

Through analysis of three years of teleretina screening across Arkansas, the prevalence of DR in patients with diabetes was 16%. Our study adds to the literature demonstrating the efficacy of telemedicine screening programs in reaching underserved populations in rural or remote counties across the United States [7–11]. A 2011 population-based study by George et al. estimated a statewide prevalence of DR at 22% [7]. Other similar screening programs in other locations in the USA found higher rates of DR in screened patients. For example, in Los Angeles, California, researchers found that 27.6% of screened patients over a one-year period had evidence of retinopathy requiring a referral to an eye care specialist [10]. Another study from the Togus, Maine Veterans Affairs Medical Center reported 23.4% of screened patients with findings of DR [11]. Though our study predicts a smaller disease burden, it still demonstrates that a substantial amount of the population requires referral to an eye care specialist.

Perceptions of the inconvenience of routine DR eye exams, such as difficulty in scheduling appointments, transport to and from appointments, lack of access to eye care providers, and competing demands on time, have been identified as a prevalent theme in patients' decisions to forgo screening [12]. Having the screening performed at the patient's routine primary care follow-up for diabetes care can mitigate some of these inconvenience barriers and increase the proportion of diabetic patients who complete regular screening. In a study incorporating DR screenings in patients' family medicine primary care visits, patients in the sample set reported that incorporating the screening into their routine care was both convenient and appreciated [13]. Though no qualitative data was collected in this study, it can be inferred from the significant proportion of patients who either did not know when their last eye exam was or had never received an eye exam that this service filled a much-needed healthcare gap.

Beyond concerns for convenience, incentives for DR screening in the primary care setting include mitigating disparities in socioeconomic and racial/ethnic populations. Studies have shown significant disparities in DR prevalence, screening rates, and treatment rates in those of lower socioeconomic status and certain minority populations, such as Native American, Black or African American, and Hispanic populations [13–18]. In our study, the group with the largest prevalence of DR was the group whose ethnicity was listed as “Other” (45%), followed by American Indian (25%). However, because only five and four patients belonging to these categories were screened through the program, no significant conclusion can be drawn about these high prevalence rates. The group with the next highest burden of DR was patients who identified as Black or African American at 19.2%. This group included 213 patients, making this prevalence rate more likely to reflect the true rate in this population. This falls in line with other literature suggesting a higher prevalence of DR in Black or African American populations. Interestingly, the prevalence of DR in patients identifying as Hispanic or Latino was low at 8.7%. This, however, could be due to the relatively small sample size in our data (23 patients).

Minority populations in our study all had a higher prevalence of other pathologies found on imaging compared to the White population. Observational studies have shown decreased rates of receiving dilated eye exams in ethnic minority populations compared to White patients for a variety of factors at the patient, provider, and system level [16, 19–22]. The lack of routine screening may explain the higher incidence rates of other pathologies found during our intervention.

The statewide telemedicine program used to screen for diabetic retinopathy in Arkansas has been a valuable resource for many patients. However, the analysis of this program also has limitations that should be considered.

One limitation of the study is that different clinics utilized different cameras to capture images of patient's retinas. This can introduce variation in the quality and resolution of the images captured, potentially leading to variability in the accuracy of the diagnoses made. Without standardization of the equipment used, it may be difficult to draw reliable conclusions about the effectiveness of the program.

Another limitation is the missing ethnicity data from 5% of the patients, which could potentially impact the accuracy of the results. Ethnicity has been identified as an important risk factor for diabetic retinopathy, with some groups being more susceptible than others. Without this data, it may be challenging to draw accurate conclusions about the prevalence and incidence of diabetic retinopathy in the population and whether the program is effective in reaching all groups equally.

Moreover, it is important to consider the potential selection bias that may have occurred in the analysis. The patients who chose to participate in the program may have differed from those who did not participate in ways that are not accounted for in the analysis, such as socioeconomic status, education level, or health status. This could limit the generalizability of the results to a broader population.

Finally, it is important to note that the study was conducted within the specific context of Arkansas and may not necessarily be generalizable to other states or regions with different populations, healthcare systems, or telemedicine programs.

Multiple potential areas for improvement were identified through our analysis. One is the need to increase utilization rates in certain areas of the state. Different regions of the state are disproportionately represented in the current sample; the East clinic site (13 patients, 1.94%), West clinic site (4 patients, 0.6%), and South Central clinic site (31 patients, 4.64%) were not well represented in the total dataset. This disparity in screening rates should inspire investigations into each of these regional centers to identify any problems or barriers preventing patient screening that can be addressed. One regional center, the South, is completely absent from the current sample as their center's retina camera has not been set up yet to begin screenings.

Another area for improvement involves the establishment of a more robust system to track follow-up exams for patients who were referred for findings on screening. This would allow for a more complete evaluation of the program.

The quality of images taken varied from clinic to clinic, most likely due to sample size and the model of the camera used. The clinic site with the largest proportion of uninterpretable images was the East clinic, which had 23.1% of its images with this designation. This may be due to the relatively smaller number of images performed at this clinic; only 13 patients to date have been screened at the East clinical site. However, other clinics with low screening numbers, such as the Family Medical Center in Little Rock, the West clinical site, and the Rahling clinical site each had no images deemed uninterpretable. Going forward, it may be prudent to track the quality of images produced from each clinical site to serve as a checkpoint on when to reassess training protocols for image capturers.

The combination of newer, easier-to-use technology and training in-house clinic staff to take images can prove to be a cost-effective method of screening patients as the process is highly time-efficient and no additional staff had to be hired.

## Figures and Tables

**Table 1:** Descriptive statistics of patients screened stratified by clinic location.

	Total (*N* = 668)	East (*N* = 13)	FMC LR (*N* = 6)	Internal medicine (*N* = 141)	North Central (*N* = 101)	Northeast (*N* = 234)	Northwest (*N* = 60)	Rahling (*N* = 15)	South Central (*N* = 31)	Southwest (*N* = 63)	West (*N* = 4)
Gender											
Male	312 (47%)	6 (46%)	3 (50%)	68 (48%)	51 (50%)	101 (43%)	32 (53%)	7 (47%)	15 (48%)	28 (44%)	1 (25%)
Female	356 (53%)	7 (54%)	3 (50%)	73 (52%)	50 (50%)	133 (57%)	28 (47%)	8 (53%)	16 (52%)	35 (56%)	3 (75%)
Age (years)											
Mean (SD)	56.15 (±13.29)	52.85 (±16.86)	61.33 (±6.802)	57.45 (±12.56)	61.51 (±13.00)	53.31 (±12.86)	57.73 (±13.74)	53.27 (±12.03)	51.39 (±14.43)	57.05 (±11.61)	53.00 (±28.66)
Ethnicity											
American Indian	4 (1%)	0 (0%)	0 (0%)	0 (0%)	0 (0%)	3 (1%)	0 (0%)	1 (7%)	0 (0%)	0 (0%)	0 (0%)
Asian	9 (1%)	0 (0%)	0 (0%)	2 (1%)	3 (3%)	0 (0%)	2 (3%)	0 (0%)	0 (0%)	1 (2%)	1 (25%)
Black or African American	221 (33%)	10 (77%)	3 (50%)	85 (60%)	3 (3%)	61 (26%)	4 (7%)	7 (47%)	24 (77%)	24 (38%)	0 (0%)
Hispanic or Latino	24 (4%)	0 (0%)	0 (0%)	3 (2%)	3 (3%)	7 (3%)	11 (18%)	0 (0%)	0 (0%)	0 (0%)	0 (0%)
Not on file	31 (5%)	1 (8%)	0 (0%)	0 (0%)	6 (6%)	20 (9%)	1 (2%)	1 (7%)	0 (0%)	2 (3%)	0 (0%)
Other	5 (1%)	0 (0%)	0 (0%)	1 (1%)	0 (0%)	1 (0%)	2 (3%)	0 (0%)	0 (0%)	0 (0%)	1 (25%)
White	374 (56%)	2 (15%)	3 (50%)	50 (35%)	86 (85%)	142 (61%)	40 (67%)	6 (40%)	7 (23%)	36 (57%)	2 (50%)
Diabetic retinopathy											
No	541 (81%)	7 (54%)	5 (83%)	113 (80%)	86 (85%)	194 (83%)	50 (83%)	11 (73%)	22 (71%)	49 (78%)	4 (100%)
Yes	104 (16%)	3 (23%)	1 (17%)	26 (18%)	12 (12%)	34 (15%)	7 (12%)	4 (27%)	8 (26%)	9 (14%)	0 (0%)
Undetermined	23 (3.4%)	3 (23.1%)	0 (0%)	2 (1.4%)	3 (3.0%)	6 (2.6%)	3 (5.0%)	0 (0%)	1 (3.2%)	5 (7.9%)	0 (0%)
Diabetes											
Type I	21 (3%)	2 (15%)	0 (0%)	4 (3%)	1 (1%)	7 (3%)	1 (2%)	0 (0%)	3 (10%)	2 (3%)	1 (25%)
Type II	647 (97%)	11 (85%)	6 (100%)	137 (97%)	100 (99%)	227 (97%)	59 (98%)	15 (100%)	28 (90%)	61 (97%)	3 (75%)
Insulin usage											
No	424 (63%)	7 (54%)	5 (83%)	86 (61%)	74 (73%)	149 (64%)	42 (70%)	12 (80%)	12 (39%)	36 (57%)	1 (25%)
Yes	244 (37%)	6 (46%)	1 (17%)	55 (39%)	27 (27%)	85 (36%)	18 (30%)	3 (20%)	19 (61%)	27 (43%)	3 (75%)
Other pathology present											
No	423 (63%)	7 (54%)	5 (83%)	78 (55%)	68 (67%)	159 (68%)	40 (67%)	6 (40%)	20 (65%)	39 (62%)	1 (25%)
Yes	245 (37%)	6 (46%)	1 (17%)	63 (45%)	33 (33%)	75 (32%)	20 (33%)	9 (60%)	11 (35%)	24 (38%)	3 (75%)

**Table 2:** Image quality by clinical site.

	East (*N* = 13)	FMC LR (*N* = 6)	Internal medicine (*N* = 141)	North Central (*N* = 101)	Northeast (*N* = 234)	Northwest (*N* = 60)	Rahling (*N* = 15)	South Central (*N* = 31)	Southwest (*N* = 63)	West (*N* = 4)	Overall (*N* = 668)
Image quality											
Adequate	5 (38.5%)	5 (83.3%)	98 (69.5%)	80 (79.2%)	167 (71.4%)	46 (76.7%)	13 (86.7%)	18 (58.1%)	34 (54.0%)	1 (25.0%)	467 (69.9%)
Blurry or missing field	5 (38.5%)	1 (16.7%)	41 (29.1%)	18 (17.8%)	61 (26.1%)	11 (18.3%)	2 (13.3%)	12 (38.7%)	24 (38.1%)	3 (75.0%)	178 (26.6%)
Unable to interpret	3 (23.1%)	0 (0%)	2 (1.4%)	3 (3.0%)	6 (2.6%)	3 (5.0%)	0 (0%)	1 (3.2%)	5 (7.9%)	0 (0%)	23 (3.4%)

FMC LR: Family Medical Center Little Rock.

**Table 3:** Descriptive statistics of those with interpretable images broken down by DM type.

	Total (*N* = 645)	Type I (*N* = 20)	Type II (*N* = 625)
Gender			
Male	300 (47%)	9 (45%)	291 (47%)
Female	345 (53%)	11 (55%)	334 (53%)
Age (years)			
Mean (SD)	56.01 (±13.25)	44.40 (±15.68)	56.39 (±13.01)
Diabetic retinopathy			
No	542 (84%)	12 (60%)	530 (85%)
Yes	103 (16%)	8 (40%)	95 (15%)
Diabetes			
Type I	20 (3%)	20 (100%)	0 (0%)
Type II	625 (97%)	0 (0%)	625 (100%)
Insulin usage			
No	413 (64%)	1 (5%)	412 (66%)
Yes	232 (36%)	19 (95%)	213 (34%)
Other pathology present			
No	401 (62%)	15 (75%)	386 (62%)
Yes	244 (38%)	5 (25%)	239 (38%)

**Table 4:** Prevalence of DR by ethnicity.

	American Indian (*N* = 4)	Asian (*N* = 9)	Black or African American (*N* = 213)	Hispanic or Latino (*N* = 23)	Not on file (*N* = 30)	Other (*N* = 5)	White (*N* = 361)	Total (*N* = 645)
Diabetic retinopathy								
No	3 (75.0%)	9 (100%)	172 (80.8%)	21 (91.3%)	25 (83.3%)	3 (60.0%)	308 (85.3%)	541 (83.9%)
Yes	1 (25.0%)	0 (0%)	41 (19.2%)	2 (8.7%)	5 (16.7%)	2 (40.0%)	53 (14.7%)	104 (16.1%)

**Table 5:** Other pathologies observed in interpretable images.

Pathology	Number of observations
Hypertensive retinopathy	71
Glaucoma suspect	70
Cataract	67
Age-related macular degeneration (exudative or nonexudative)	17
Optic nerve/disc edema (unilateral or bilateral)	17
Choroidal nevus	14
Macular scar	6
Chorioretinal scar	4
Hollenhorst plaque(s)	4
Retinitis pigmentosa	4
Optic nerve pallor	4
Retinal detachment: present or evidence of prior	3
Venous occlusion/insufficiency	3
Optic nerve hemorrhage	2
Macular granular deposits	1
Macular hole	1
Presumed ocular histoplasmosis syndrome	1
Pigment epithelial detachment	1

**Table 6:** Other pathology present by ethnicity.

	American Indian (*N* = 4)	Asian (*N* = 9)	Black or African American (*N* = 221)	Hispanic or Latino (*N* = 24)	Not on file (*N* = 31)	Other (*N* = 5)	White (*N* = 374)	Total (*N* = 668)
Other pathology present								
No	2 (50.0%)	4 (44.4%)	110 (49.8%)	15 (62.5%)	23 (74.2%)	2 (40.0%)	267 (71.3%)	423 (63.3%)
Yes	2 (50.0%)	5 (55.6%)	111 (50.2%)	9 (37.5%)	8 (25.8%)	3 (60.0%)	107 (28.6%)	245 (36.6%)

## Data Availability

The data used to support the findings of this study are restricted by the UAMS Institutional Review Board to protect patient privacy. Data are available by contacting the corresponding author for researchers who meet the criteria for access to confidential data.
